# Metastatic ethmoidal alveolar rhabdomyosarcoma presenting as a scalp nodule in an adult woman

**DOI:** 10.1016/j.jdcr.2022.02.010

**Published:** 2022-02-26

**Authors:** Nathan Chow, Jeremy Purser, Cloyce Stetson, Ashley Sturgeon

**Affiliations:** a School of Medicine, Texas Tech University Health Sciences Center, Lubbock, Texas; b Department of Dermatology, Texas Tech University Health Sciences Center, Lubbock, Texas

**Keywords:** alveolar rhabdomyosarcoma, cutaneous metastasis, rhabdomyosarcoma, scalp nodule, ARMS, alveolar rhabdomyosarcoma, RMS, rhabdomyosarcoma

## Introduction

Rhabdomyosarcoma (RMS) is a soft-tissue sarcoma of mesenchymal origin that exhibits skeletal muscle differentiation. It is classified into various histologic subtypes, including alveolar rhabdomyosarcoma (ARMS), embryonal rhabdomyosarcoma, pleomorphic rhabdomyosarcoma, and sclerosing rhabdomyosarcoma.^1^ RMS is the most common soft-tissue tumor in children and adolescents, constituting more than 50% of soft-tissue sarcomas. However, RMS in adults is extremely rare and associated with poorer outcomes.^2^ We present the case of a 67-year-old woman with metastatic right ethmoidal ARMS manifesting as a scalp nodule.

## Case report

**Fig 1:**
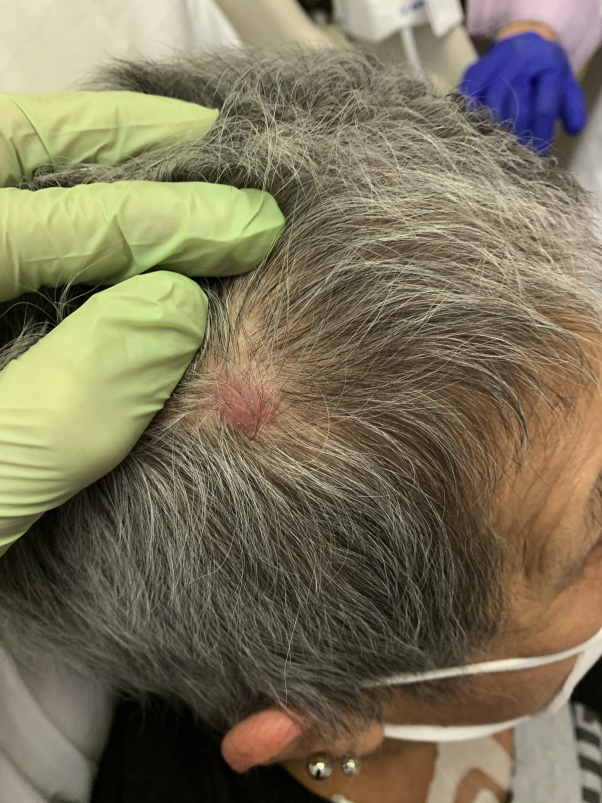
Clinical image of the scalp nodule before biopsy.

A 67-year-old woman admitted for inpatient chemotherapy for ARMS presented with a scalp nodule. The patient noticed the enlarging, asymptomatic nodule 3 months ago on the parietal aspect of the right side of the scalp (Fig 1). She denied any history of similar lesions. She had been diagnosed with right ethmoidal ARMS 9 months previously after experiencing months of sinus symptoms, facial swelling, numbness, and pain. Her symptoms worsened to right-sided vision difficulties and occipital head and neck pain. She presented to the emergency department where magnetic resonance imaging of the orbits showed an enhancing mass with the epicenter in the right ethmoid air cells, with extension into the right side of the maxillary sinus, right side of the orbital apex, anterior aspect of the right side of the cranial fossa, middle aspect of the right side of the cranial fossa, and right side of the cavernous sinus. Otolaryngology performed a right maxillary antrostomy, ethmoidectomy, and sphenoidotomy. Pathologic analysis of surgical specimens revealed diffusely infiltrative neoplasm composed of clusters and sheets of small round neoplastic cells. Immunohistochemical staining was positive for desmin, myoD1, and myogenin. A PAX3-FOXO1 fusion was identified on a gene fusion panel. Altogether, these findings were consistent with a diagnosis of ARMS. Computed tomography of the chest, abdomen, and pelvis 9 months previously had revealed no evidence of metastatic disease. She received 2 rounds of a chemotherapy regimen consisting of vincristine, cyclophosphamide, and doxorubicin alternating with ifosfamide and etoposide. After completing the second round 4 months previsouly, magnetic resonance imaging of the orbits showed significant resolution of the previously identified mass. Radiotherapy was then performed 2 months later. Another magnetic resonance imaging of the orbits was ordered 1 month previously, which showed a 9 × 6-mm focus of abnormal enhancement and abnormal restricted diffusion in the frontal aspect of the right side of the scalp.

**Fig 2:**
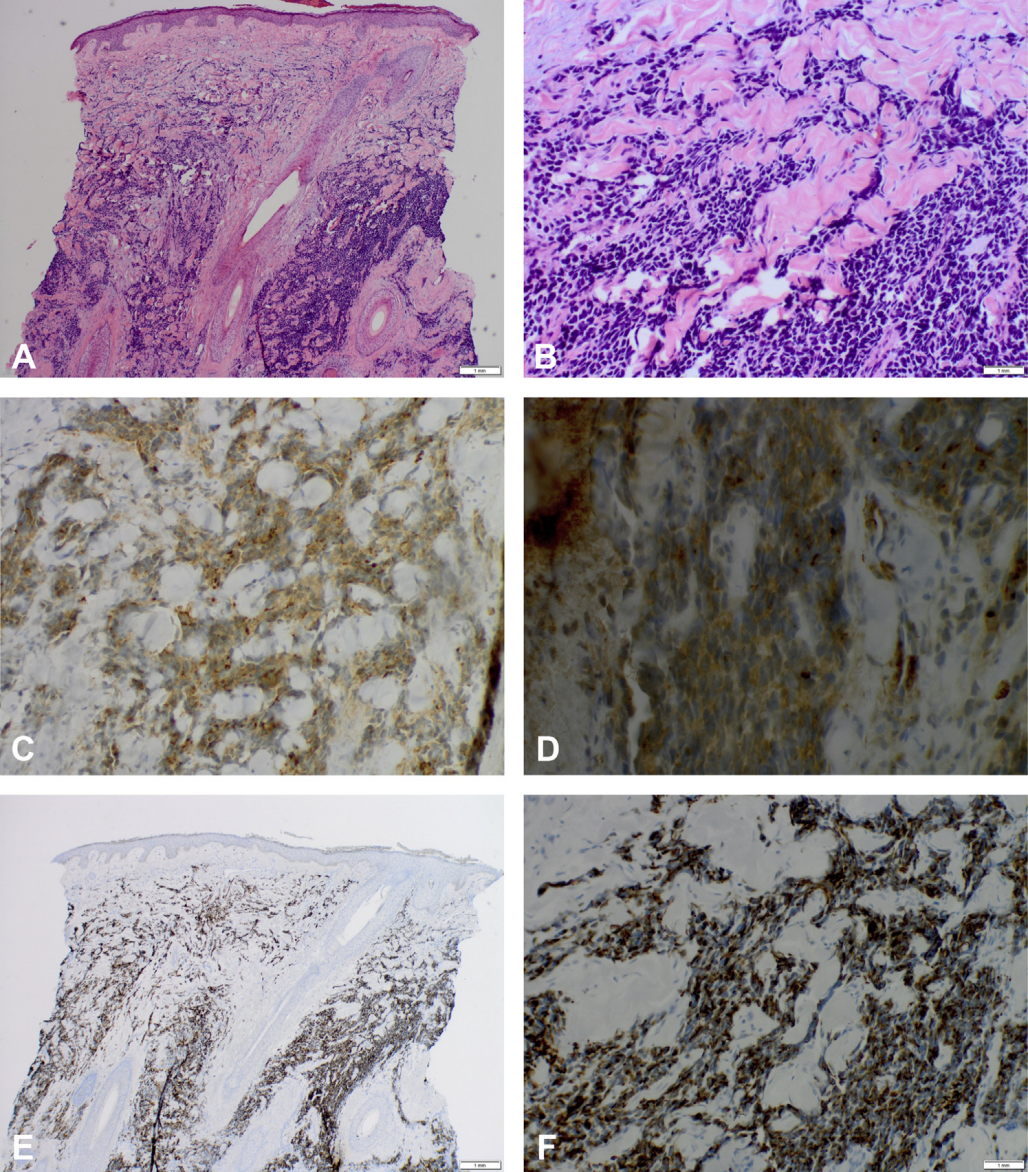
**A** and **B,** Low-magnification and high-magnification images of hematoxylin and eosin staining, revealing malignant tumor composed of micronodular aggregates of small blue cells. **C** and **D,** Low and high magnification of strongly positive muscle-specific actin staining. **E** and **F,** Low and high magnification of strongly positive desmin staining. (**A** and **B,** Hematoxylin and eosin staining; original magnifications: **A,** ×40; **B,** ×200; **C** and **D,** muscle-specific actin staining; original magnifications: **C,** ×100; **D,** ×200; **E** and **F,** desmin staining; original magnifications: **E,** ×40; **F,** ×200).

Physical examination revealed a 15-mm mobile, erythematous nodule on the parietal aspect of the right side of the scalp. A 4-mm punch biopsy revealed malignant tumor composed of micronodular aggregates of small blue cells with strongly positive for muscle-specific actin and desmin (Fig 2), consistent with a histologic diagnosis of metastatic ARMS.

Three months later, otolaryngology performed a wide local excision of the mass which had enlarged to 4.7 × 2.0 cm. A 2.5-cm mass *on the* *right side of the buccal mucosa* was also identified; an incisional biopsy demonstrated histologic findings consistent with metastatic ARMS. Further treatment and next steps are to be determined by oncology.

## Discussion

In the United States, soft-tissue sarcomas account for 1% of adult malignancies, of which 2% to 5% can be attributed to adult RMS.^3^ The disease has a slight male predominance and can present as a primary malignancy or, rarely, in association with neurofibromatosis type 1.^1^ Among the 4 variants, embryonal and alveolar RMS are more common in children, whereas less so in adults.^4^ The alveolar variant, named after its alveolar pattern on histology, is the second most common subtype and typically presents in the deep tissues of the extremities.^1^^,^^3^^,^^5^ ARMS has poor survival rates due to higher rates of metastasis, with 1 study reporting a 25.9% rate of metastasis.^5^ Furthermore, cutaneous metastasis of adult RMS is exceedingly rare. A review of the literature identified 4 adult cases of RMS with cutaneous metastasis to the head, neck, trunk, face, and lip; only 1 individual was above the age of 65.^6^^,^^7^

It is well documented that adults with RMS have poorer outcomes compared with children.^2^^,^^4^ Sultan et al^4^ reported 5-year survival estimates of 82% in children with localized disease but only 47% in adults with localized disease. The reasons behind this difference are unknown; suggestions include multidrug-resistant proteins in adult disease or lower tolerance of adults to intensive therapy.^4^ In all ages, treatment failure is typically due to drug resistance and metastatic disease.^1^ Favorable prognostic factors include age ≤20 years, tumors ≤5 cm, absence of regional or distant disease, and surgical resection with negative margins.^3^ Patient age is an independent prognostic variable in adults and children,^3^ and a progressive decline in survival curves was noted with advancing age.^4^ Multimodal therapy with surgery, radiotherapy, and chemotherapy has improved survival in pediatric patients to nearly 70%. Those with localized disease have significantly improved survival rates, but the prognosis has not improved significantly in those with metastatic disease.^8^ In adults, no controlled, prospective trials assessing treatment have been performed due to the rarity of the disease. Generally, adult RMS can be treated similarly to pediatric RMS with a multimodal regimen.^1^^,^^2^ However, this has not improved overall survival rates in adults, which are estimated to be between 20% and 40%.^2^

Generally, RMS presents as a fleshy-lesion, erythematous mass with overlying telangiectasia, or soft-tissue swelling.^9^ The clinical differential diagnosis for scalp nodules and other dermal or subcutaneous growths includes cysts, infection, lipoma, primary skin tumors (eg, basal cell carcinoma, squamous cell carcinoma, Merkel cell carcinoma, and lymphoma); metastatic disease, such as breast cancer, or other sarcomas such as liposarcoma.^10^ The histologic differential diagnosis includes lymphoma, Merkel cell carcinoma, neuroblastoma, and Ewing sarcoma. These were ruled out in our patient upon histologic and immunohistochemical analysis. Histology findings of ARMS typically include undifferentiated, small, round cells growing either in a pattern resembling pulmonary alveoli or diffuse sheets with immunoreactivity for desmin, muscle-specific actin, and myogenin.^3^^,^^4^

In summary, we report a case of metastatic ethmoidal ARMS presenting as a scalp nodule in an adult woman. Our case adds to the existing literature regarding the clinical and histopathologic findings of cutaneous metastasis from RMS. Although rare, scalp nodules or other cutaneous lesions in individuals with soft-tissue malignancy may represent metastasis and necessitate biopsy to avoid misdiagnosis. Confirmation of metastasis may warrant referral to oncology for further work-up of tumor spread.

## Conflicts of interest

None disclosed.
